# Radiation Processing of Styrene-isoprene-styrene/Poly(ε-caprolactone) Blends

**DOI:** 10.3390/polym14214737

**Published:** 2022-11-04

**Authors:** Eduard-Marius Lungulescu, Traian Zaharescu

**Affiliations:** National Institute for Research and Development in Electrical Engineering ICPE-CA, 313 Splaiul Unirii, 030138 Bucharest, Romania

**Keywords:** SIS, PCL, γ-irradiation, compatibilization, chemiluminescence, DSC, FTIR

## Abstract

The irradiation consequences on styrene-isoprene-styrene (SIS)/poly(ε-caprolactone) (PCL) blends are discussed starting from the oxidation initiation. Three characterization methods: chemiluminescence, differential scanning calorimetry and FTIR spectroscopy are applied. The differences that exist between the two components are revealed, when the oxidation rates of the inspected formulas depend on the blending proportion and the degradation conditions. The relevant activation energies characterizing the oxidation strength as well as the kinetic parameters of degradation during the accelerated damaging of blended polymers are related to the inhibition protection of PCL on the faster oxidation of SIS. The interaction between mixed components is revealed by the structural modifications simultaneously accompanied by the competition of formation and decay of radicals.

## 1. Introduction

The radiation processing of polymer blends represents a convenient procedure by which the components are intimately compatibilized and the blend gains the foreseen service performances [1]. The stability of this kind of material system is an essential characteristic that recommend them for different appropriate applications [2]. The radiation processing of polymer formulations allows the conversion of physical mixtures into homogenous materials, where the components are intimately linked [3]. The fragmentation of macromolecules, the decay of free radicals by recombination, the crosslinking of macroradicals or coupling of chains into stable structures by functional monomers are the main steps that define the mechanisms of polymer blends modelling [4]. Apart from the improvement of consistency, the delay of oxidative degradation must be reached by the increase in the number of intermolecular bridges [5], the addition of proper stabilizer [6] or the presence of appropriate filler [7,8]. An illustrative example for the mitigation of degradation in γ-exposed polymers is the study on the irradiation effects in styrene-butadiene matrix [9], where the material properties are analyzed starting from the reactions of intermediates appeared during radiolysis.

The development of polymer areas by the structuration of their blends engages the mixing of various types of polymers, whose degradation mechanisms are complementary, even though they are less similar. The compatibilization of components is successfully attained by the exposure to high-energy radiation (accelerated electron beams or γ-rays), which guarantee the interpenetration and crosslinking [10,11]. This process can be achieved by the energy transfer [12] followed by the recombination of free radicals spread in the material. The inhibition of oxidative degradation is a main requirement in the high-energy treatments, because the concentrations of reactive entities are great and the probability of their reaction with diffused oxygen can’t be discarded. Otherwise, the propagation of oxidation deteriorates the material quality and limits the durability [13]. Starting from the ideas on polymer fragmentation, the modification occurred in the properties of polymer blends can be conducted onto the desired goal [14].

The radiation processing of polymer blends consisting of component differing by the radiochemical behaviors is an attractive method for the preparation of materials with the expected properties [15,16,17]. The improvement of functional properties by high-energy exposure is certainly obtained by the component compatibilization, when the free radical source is one of the mixing components [18]. The reactivity of radicals is the basic reason by which the mixed components are jointed to each other. The development of the polymer network is assisted by the accomplishment of the recombination reactions, which may include the decay of the most abundant radicals. The lifetime of radiation processed materials is correlated with the development of crosslinking, which maintains the material integrity over the hard condition periods [19]. The competition between crosslinking and scission that characterizes the evolution of polymer energetic treatment forwards the material stability in relation with the new developed structure. The processing mechanism involving former molecular scissions, migration of radicals, recombination and disproportionation, oxidation indicates the latest molecular configuration that finally defines the application ranges [20]. The achievement of conversion onto the stable material is confidently based on the contributions of blending components, which provide intermediates for their structuring in the new ordered fragments [5].

When a component of polymer mixture does not follow a classical mechanism of structuring on new molecular configuration, like poly(lactic acid) or poly(ε-caprolactone), the rearrangement of damaging fragments follows the recombination way of the chain propagation, which takes place as a self-supporting process [21]. The random reactions of free radicals involve the participation of all types of intermediates that includes the carbon and oxygen centered moieties. The contribution of the weakest component of blends determines the radiation behavior of processing [22]. Simultaneous competition between the recombination of radical intermediates and their tendencies onto oxidation limits the stability of processed material which strongly influence the post-irradiation properties [21]. The conversion of polymer blends into potential applicable materials follows the gold role of jointing molecular fragments appeared from the scission of less stable component on the backbone where radical positions allow the rebuilding of new structure [22].

The availability of free radicals is the reason by which the progress in their reaction chains is related to the anticipated and controlled structuration [23,24]. The inclusion of poly(ε-caprolactone) by homogenization into the SIS phase increases the probability of interphase reactions, which may conduct the processed material to a higher resistance against oxidation [25]. The modifications achieved during the high energy exposure treatments (electron beam irradiations) of PCL are related to the decrease in the molecular weight rather than to the material oxidation. The experimental proofs obtained in the blends consisting of PCL and PLA, the measurements of melt flow rate, mechanical properties and morphology [26], provide the importance of mixing proportion, which certifies the relative convenient stability of PCL in respect with PLA. The confirmation of the crosslinking potential was obtained by the addition of TAIC (triallyl isocyanurate) for the efficient curing of PCL [27] as the source of active radicals.

The previous insight on a polymer couple consisting of PLA and SIS [28] reveals the influence of PLA playing the role of radical provider for the formation of the material stabilized fraction. In the case of SIS/PCL systems, the two components are the sources of radicals centered on oxygen atoms (PCL or PLA) and the second one centered on carbon atoms (SIS).

The present work analyzes the evolution of degradation stability as the measure of curing by means of the chemiluminescence, DSC and FTIR methods. The modifications in the number of emitted photons showing how the degradation progresses illustrate the manner through which the two components, SIS and PCL, provide and accept radicals. The proportion between these two contradictory processes, scission and recombination, is described by the values of chemiluminescence intensities, which varies in relation with the degree of oxidation [20]. The novelty of this study is based on the association between the two types of polymers, one of them being biomaterial. The studied blends may be destined to the manufacture of various ecological packaging materials presumable sterilized by ionizing radiation or may be considered as an example for the polymer recycling by radiation processing. The extension of stability investigation is useful for the identification of the procedure through which a certain blending ratio is the appropriate composition with a relevant durability.

## 2. Materials and Methods

The polymer materials used in this study are styrene-isoprene-styrene block copolymer (SIS) manufactures by KRATON (USA) as D1165 PT sort. This material was purchased as (1,7)-polyoxepan-2-one pellets with an average diameter of ~3 mm. The styrene content is 30 wt%, density 1.145 g mL^−1^ @ 25 °C, average M_n_ 80,000 and the polydispersion index (M_w_/M_n_) is less than 2. Poly(ε-caprolactone) provided by Sigma Aldrich (USA) having average weight molecular weight M_w_ ~14.000 and average numerical molecular weight M_n_ ~14.000.

For the preparation of samples each polymer was dissolved in chloroform and the final solutions had the concentration of 1 g mL^−1^. The three blends (3:1, 1:1 and 1:3) were prepared by the dissolution of components in the proper adjusted amounts and the final solutions have the same concentration of 1 g mL^−1^. All the samples destined to the chemiluminescence determinations were prepared by pouring appropriate solution into small round aluminum pans (diameter 6 mm) for the evaporation of solvent. The drying was accomplished at room temperature when thin films (thickness 10 µm) were obtained. The samples investigated by other methods (DSC and FTIR) were obtained in the same manner, but the diameter of the pan was 15 mm and the film thickness was 0.5 mm).

The chemiluminescence determinations are based on the emission of a photon for each excited carbonyl formed by the reaction between one free radical and diffused oxygen molecules during oxidation. The proportionality between the number of oxidized radicals and the number of counted photons allows the description of the evolution of degradation either at a continuous heating at constant temperature (isothermal method) or over a large temperature range (nonisothermal method), when the rising temperature induces molecular scission and the formation of oxygen-containing structures.

The γ-exposure was accomplished in air, in an irradiation machine (Ob-Servo Sanguis, Hungary) provided with ^60^Co sources, at four integrated doses of 0, 25, 50 and 100 kGy by permanent rotation of irradiation can. Irradiation of polymer samples was carried out at a dose rate of 0.6 kGy h^−1^. The dose accumulation procedure was preferred because it preserves the identity of the sample. Both control and blend samples were investigated immediately after the end of each irradiation minimizing the effects of storage.

Differential scanning calorimetry (DSC) measurements were performed on SETARAM 131 EVO (Setaram Instrumentation, France) under the following conditions: temperature range from 30 °C to 340 °C, heating rate, 10 K·min^−1^, atmosphere—air with gas flow of 50 mL·min^−1^. Samples of about 4–7 mg were measured in aluminum pans of 30 μL. Different parameters characterizing the observed effects, such as melting temperatures (*T_m_*) and their associated thermal effects (*ΔH_m_*) and OOT *(Onset Oxidation Temperature)* values were determined from the DSC curves.

The degree of crystallinity of SIS-PCL blends was determined with the equation [29]:

$$\mathrm{Crystallinity}\left(\%\right)=\;\frac{{\Delta H}_{m}}{{\Delta H}_{m}^{0}}\cdot 100$$

where *ΔH_m_* is the enthalpy of fusion measured at the melting point; $\Delta H_{m}^{0}$ is the enthalpy of fusion of 100% crystalline PCL (i.e., 139.5 J/g [29]).

The Fourier transformed infrared spectra (FTIR) were recorded on a Jasco FTIR -4200 (Jasco, JP), coupled with and Attenuated Total Reflectance (ATR) Jasco PRO 470-H module, between 650–4000 cm^−1^, at a resolution of 4 cm^−1^ and 50 scans/spectrum.

## 3. Results and Discussion

The structural modifications induced by the exposure of polymers to γ-radiation can be watched by the evolution of material responses, which indicate the detailed information on the processing routes. Due to the great differences between the scission manners for these two polymers, SIS and PCL, the unlike behavior of their blends is shown during the selected investigations. The interactions between degradation intermediates lead to the specific concern by which the reorganization of fragments provides appropriate answers.

### 3.1. Chemiluminescence

The measurements of chemiluminescence emission intensities allow the identification of fundamental changes occurred in the SIS/PCL blends as the result of the individual contribution of components by the reactions of the bearing free radicals [28]. Both components may be fragmented, but SIS fraction is more unstable in respect with PCL [30]. The chemiluminescence measurements make possible the watching the formation and the decay of radical fate, the entities involved in the degradation route. The increasing CL intensity indicates the more advanced oxidation by the enhanced number of photons, while the diminution of emission intensity shows the consumption of radicals by oxidation or recombination, when the stability of material reaches a steady state of a final stage of the process.

#### 3.1.1. Isothermal Chemiluminescence

**Figure 1:**
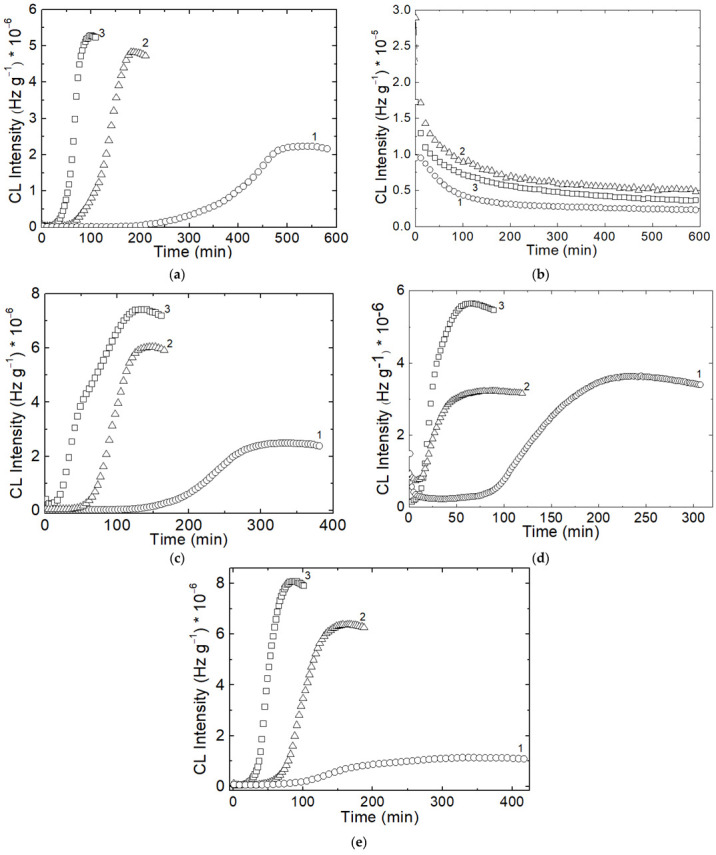
The isothermal CL spectra recorded on the unirradiated components and their blends: (**a**) neat SIS; (**b**) neat PCL; (**c**) SIS:PCL = 3:1; (**d**) SIS:PCL = 1:1; (**e**) SIS:PCL = 1:3; Testing temperatures: (1) 130 °C; (2) 140 °C; (3) 150 °C.

The triad isothermal curves for all tested compositions (Figure 1) where one component is SIS present a sigmoidal shape that proves the self-catalytic oxidative degradation like polyolefins [31]. This comportment influences the related to the loading of this polymer by the generation of the fragments capable to scavenge the other similar moieties or the radicals supplied by the scission of PCL molecules. The recording of lower values of CL intensities for the mixture SIS:PCL = 1:1 defines the simultaneous availabilities of both components, but the susceptibility of SIS to scission would be rather relevant. The descendant shape of isothermal curves for PCL certifies the minimal role of oxidation in respect with molecular scission during CL measurements. The essential information about the thermal stability of SIS/PCL blends is obtained by the comparison of the isotherms recorded at the lowest temperature.

The medium applied temperature (140 °C) becomes the proper evidence for thermal stability of these blends, whose heights and blendings depict the oxidation strengths related in respect with sample formulations.

The hydrocarbon component (SIS) is the susceptible to breaking followed by oxidation, while the polyester (PCL) provides free radicals ended with an oxygen atom. These mechanistic arguments are the basic supports for the evident differences between the records for the two blending components.

The values of activation energies (Table 1) characterize the thermal resistances of investigated blends. As it was expected from the isothermal CL determinations, the most stable is the 1:1 blend, where poly(ε-caprolactone) plays a double role: the source of radicals and the restricting factor for oxidation propagation by its decomposition to lactide. This explanation is based on the radiation stability of components, where the polyesters present a somewhat lower resistance under the action of ionizing radiation [32]. This behavior is explained by the existence of oxygen centered radicals which are not further oxidized and the degradation chain is interrupted. These radicals become either the generator of other hydrocarbon radicals by the abstraction of protons from SIS molecules or by the recombination with the SIS fragments. The ESR study on irradiated PCL [33] indicates the value of 2.5 kGy (a low threshold) for crosslinking dose followed by a further stage of irradiation, when the competition between scission and crosslinking leads to a fast degradation.

The γ-exposure of blended components is a radical source by the fragmentation of macromolecules. While the weakest points for scission in SIS are double bonds and highest substituted carbon atoms, the polar ester bonds (–OCO–) are vulnerable points in PCL. Accordingly, the most fraction of radicals appeared from the broken SIS are converted into oxygen-containing radiolysis products, while the radicals formed by the division of PCL are not attacked by diffused oxygen and they continue the degradation by the interaction with neighbouring intermediates.

**Figure 2:**
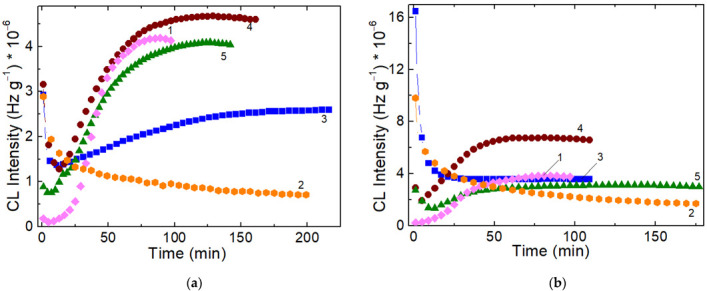
The isothermal CL spectra recorded on irradiated SIS/PCL samples at (**a**) 25 kGy and (**b**) 50 kGy. (1) neat SIS, (2) neat PCL, (3) SIS/PCL 1:3, (4) SIS/PCL 1:1; (5) SIS/PCL 3: 1. Testing temperature: 140 °C.

The degradation of studied systems, achieved by γ-irradiation, is investigated by the CL measurements at the low doses (25 kGy and 50 kGy) (Figure 2); the 25 kGy dose is characteristic for radiation sterilization. The difference for SIS and PCL in the curve shapes is also abided.

While the high values of CL intensities for the samples subjected to 50 kGy are higher because the radical concentration is greater, the advance in the degradation process is somewhat unlike. The degree of scission related to the received doses delimits the contribution of each component on the propagation of oxidation. While the neat polymers show the similar difference as they exhibit for non-irradiated samples, the blend consisting of three parts of PCL (Figure 2, curves 3) get the analogous shape with pristine PCL. It means that the fragmentation of PCL is predominant. The surprising feature of curve 4 from Figure 2 is the most advanced oxidation in the strike contrast with thermal behavior, when it exemplifies the greatest thermal stability (Table 1). The presence of PCL in the studied blends is confirmed by all isothermal CL curves recorded at 50 kGy, when the former parts of curves are like the PCL curves than the SIS ones.

**Figure 3:**
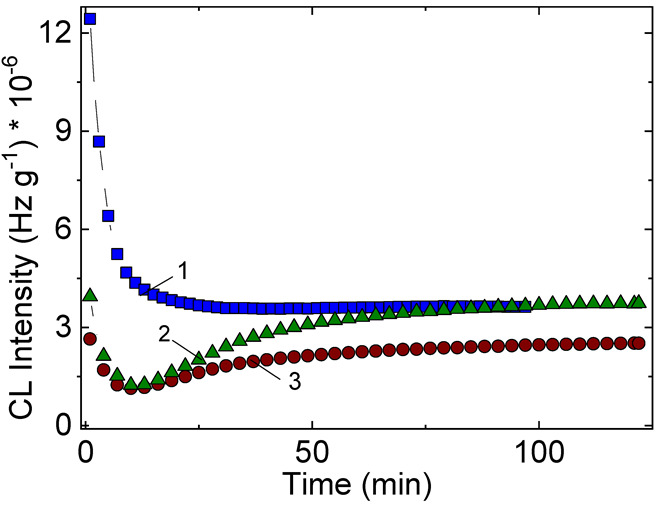
Isothermal CL spectra recorded on the samples consisting of SIS and PCL components subjected to an irradiation γ-dose of 100 kGy: (1) SIS:PCL = 3:1, (2) SIS:PCL = 1:1, (3) SIS:PCL = 1:3. Testing temperature: 140 °C.

The influence of the degradation of SIS/PCL blends is well depicted by the isothermal CL curves (Figure 3).

The fast trend of curves onto the steady state of oxidation process depends on the relative concentrations and on the degree of conversion of intermediated into oxidation products.

#### 3.1.2. Non-Isothermal Chemiluminescence

**Figure 4:**
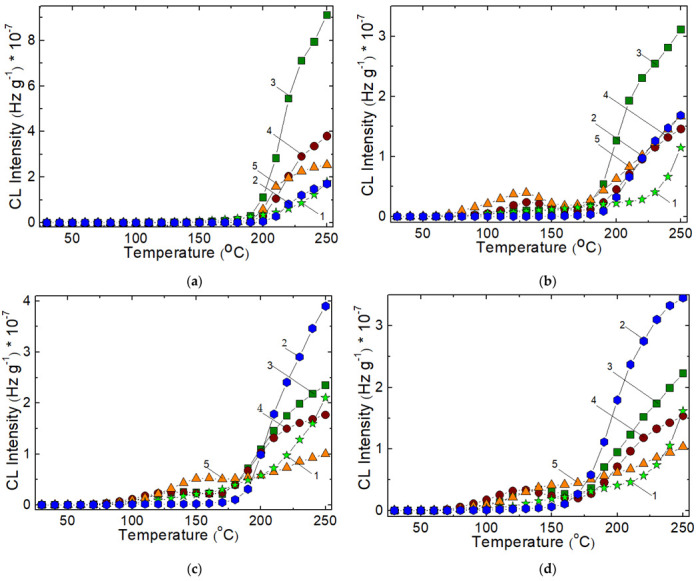
Nonisothermal CL spectra recorded on the samples consisting of SIS and PCL exposed to γ-rays at various doses: (**a**) 0 kGy, (**b**) 25 kGy, (**c**) 50 kGy, (**d**) 100 kGy; (1) neat PCL, (2) neat SIS,(3) PCL:SIS = 3:1, (4) PCL:SIS = 1:1, (5) PCL:SIS = 1:3. Heating rate: 10 °C min^−1^.

The evolutions of oxidation in respect with the rising temperature present a fundamental difference between the non-irradiated and γ-exposed samples. The presence of shoulders at various temperatures is ascribed to the formation and decay of hydroperoxides during radiolysis. The greatest heights at 125 °C for 25 kGy, at 150 °C for 50 kGy and 100 kGy appeared in the CL curve of PCL:SIS = 1:3 indicate that the hydrocarbon is the source of free radicals which are converted into hydroperoxides according with the classical mechanism of oxidation [20]. When the degradation dose rises to 50 and 100 kGy, the SIS component is strongly damaged and the blends are earlier aged. The PCL component has a slow degradation level when the temperature increases. The relatively constant intensity in the region of sustained degradation of SIS occurs, proves its minimal contribution at higher doses. While the blend prepared by blending of PCL:SIS = 1:1 proportion present a modest effects on the progress of oxidation, the PCL:SIS = 3:1 mixtures are sharply oxidized at the temperatures exceeding 180 °C for lower irradiation doses (0 and 25 kGy) and the smooth oxidation rate may be noticed for the other two applied doses (50 and 100 kGy) (Figure 4).

The advance of degradation in the samples differently irradiated occurs dissimilarly. While the samples receiving 25 kGy show an initiation of degradation by means of hydroperoxides (the peak around 130 °C), the other specimens display the flatting zones where the hydroperoxides are rather consumed that generated.

**Figure 5:**
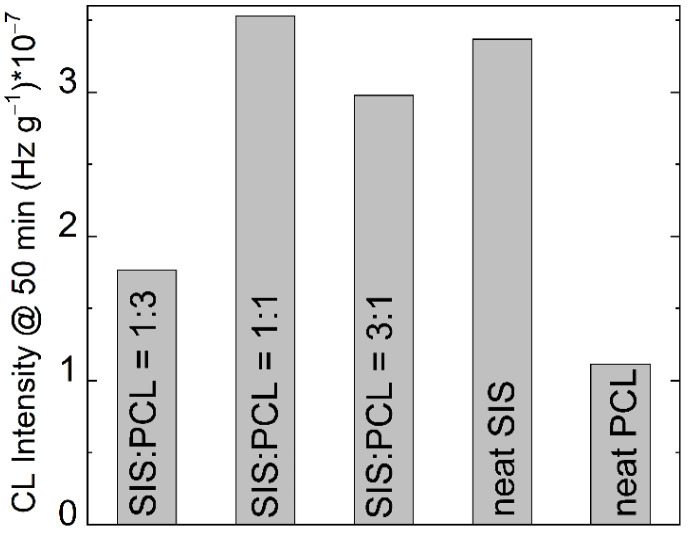
Histogram on the oxidation stages for PCL/SIS mixtures irradiated at 25 kGy.

The oxidation of SIS/PCL samples, accelerated by γ-treatment, is caused by reaction of free radicals predominantly supplied by the scissions of SIS molecules. These intermediates interact with their neighbours leading to the restructured phase by recombination or to the degraded phase by reactions with diffused oxygen. As it may be noticed from Figure 5, the participation of SIS is more evident in the 1:1 blend, when the propagation chain involved in oxidation is insured by the high local concentration of intermediates. Even though a small proportion of crosslinked islands appear, the initiation of oxidation is really done, when the somewhat inert intermediated from PCL surround these oxidation precursors.

**Figure 6:**
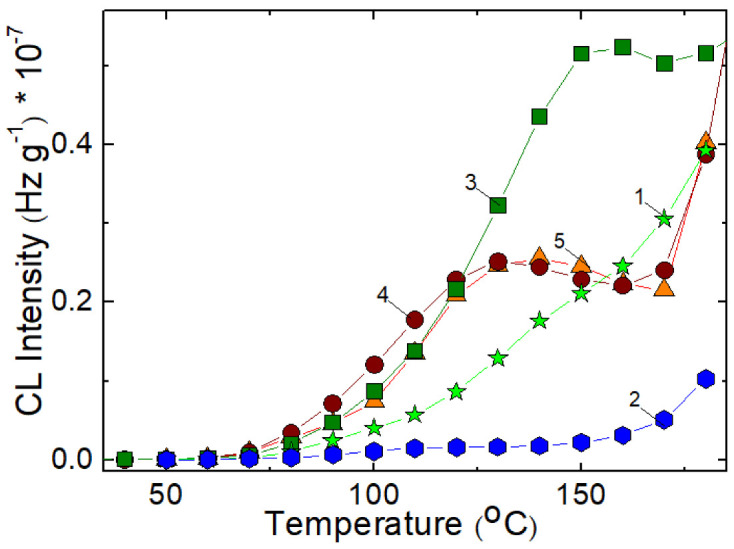
The development of oxidation on the low temperature range for PCL/SIS samples irradiated at 50 kGy: (1) neat PCL, (2) neat SIS, (3) PCL:SIS = 3:1, (4) PCL:SIS = 1:1, (5) PCL:SIS = 1:3.

**Figure 7:**
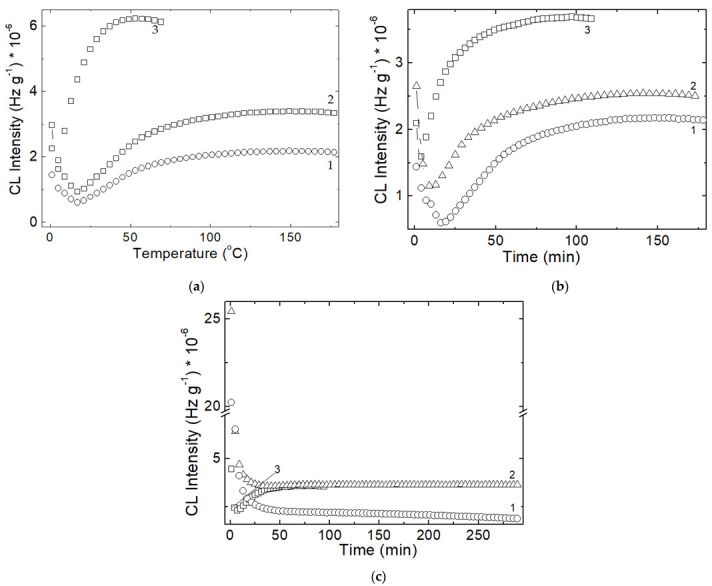
Isothermal CL spectra recorded on PCL:SIS samples after their irradiation at 100 kGy. Temperatures: (1) 130 °C; (2) 140 °C; (3) 150 °C; (**a**) PCL:SIS = 3:1; (**b**) PCL:SIS = 1:1; (**c**) PCL:SIS = 1:3.

The most important factor that dominates the advance of degradation in the studied blends is the relative radiochemical stability of the two components. From Figure 6 some outlines may be touched: the scission followed by oxidation starts much earlier in the PCL fraction than in SIS portion; this feature would determine the initiation of degradation at 60—65 °C in the investigated PCL/SIS systems; on the higher temperature range, the presence of PCL brings about a progressive advance of oxidation probably due to the action of ester intermediates on the unsaturation content in isoprene portion. The sample containing SIS as a major component is oxidized in the large extent because this polymer is much vulnerable; its structure contains two possible breaking points: tertiary carbon atoms in the styrene segments and methyl in the adjacent position in respect with double bond inside the isoprene moieties. This stability peculiarity can be easily observed for all doses (see Figure 4); Under the fast degradation caused by γ-exposure, the characteristic conditions for radiation sterilization, radiation processing of medical wear, food packaging or commodities, the behavior of PCL:SIS = 1:3 is totally unlike (Figure 7). While the blends with high and medium loadings present two distinct degradation steps, the first one is characterized by the decrease of CL intensity like PCL, the next stage of degradation presents increasing CL intensities, which depicts the behavior of SIS. The composition with the greater content of SIS exhibits the monotone decrease of CL intensities at 130 °C and 140 °C, but a curve with a minimum at 150 °C. These attributes suggest the specific contributions of components, when the applications are done under the action of high intensity stressors, for example exposure to sun light (photodegradation), overheating regime (accidental exposure to heat or fire), electrical discharge (breaking down of electrical insulation) or intense compression/detention multicycles.

### 3.2. Differential Scanning Calorimetry

DSC is one of the most versatile techniques for characterizing the thermal behavior of polymeric materials (neat, composites and blends). The technique allows the determination of phase transitions (i.e., melting, solidification, evaporation), phase separations (blends, copolymers), determination of the degree of crystallinity, determination of the effects induced by the use of additives (polymers, fillers, plasticizers, stabilizers), monitoring the effects of aging and determining the thermal stability of polymers (OIT, OOT), etc. [34,35].

**Figure 8:**
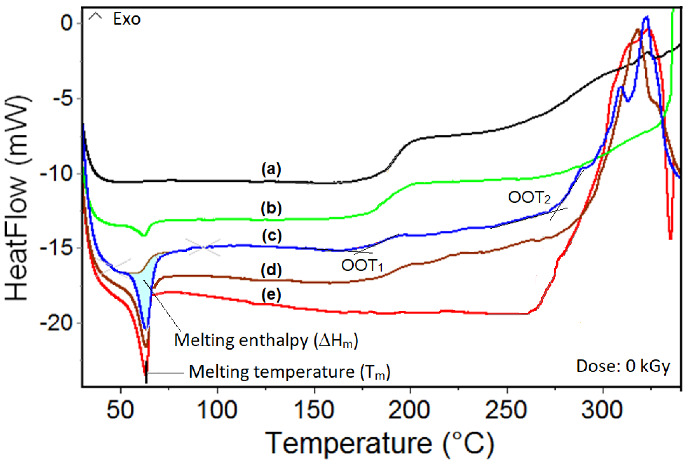
Non-isothermal, DSC curves recorded on: (a) Neat SIS; (b) SIS:PCL = 3:1; (c) SIS:PCL = 1:3;. (d) SIS:PCL = 1:1; (e) Neat PCL.

Figure 8 shows the non-isothermal DSC curves obtained on the samples of neat SIS and PCL and their blends. The variation of the DSC parameters, depending on the irradiation dose and the concentration of SIS in the blends: melting temperatures (Tm) and their associated enthalpies, for all analyzed samples, are listed in Table 2. The analysis of the DSC curves shows that the samples containing PCL present an endothermic melting peak, with the maximum at approx. 63.4 °C, which confirms its semi-crystalline structure, while SIS does not present such a peak. The maximums of the melting temperature seem to be independent of the irradiation dose for all samples containing PCL. By adding SIS, these maximums are slightly shifted towards lower melting temperatures, suggesting the immiscibility of two components. A decrease in fusion enthalpy from 39 J/g for neat PCL to 8.6 J/g for SIS:PCL = 3:1, is also noticed. It is believed that SIS, by its amorphous structure, can induce a certain degree of disorder in the semi-crystalline structure of PCL (i.e., less PCL turns completely into a crystalline state) [36,37].

**Figure 9:**
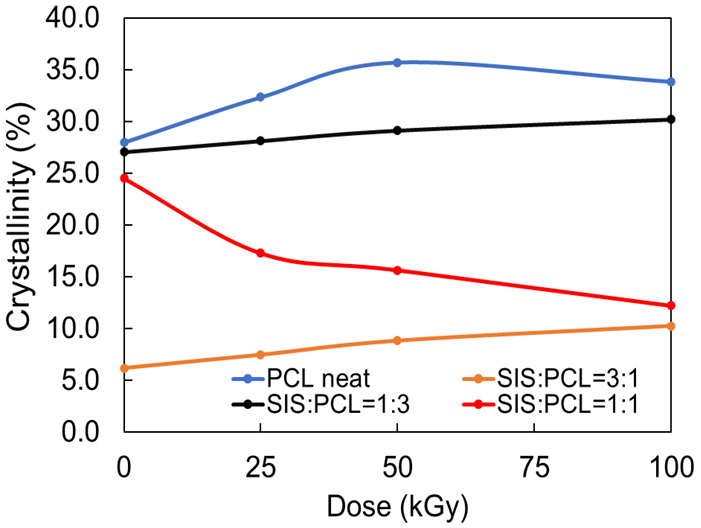
Variation of PCL crystallinity with both SIS concentration and irradiation dose.

The variation of crystallinity degree of the neat and blend samples, as a function of irradiation dose is depicted in Figure 9. A decrease in crystallinity degree with the increase in the content of SIS in blends can be observed, sustaining the disruptive effect on the PCL structure.

However, a general increase of the crystallinity of SIS/PCL blends with irradiation dose can be observed, except for SIS:PCL = 1:1 where the effect is reversed. It is known that gamma radiation processing induces radicalic processes of scission, cross-linking and oxidation [38] in the exposed materials. PCL is a polymer with a semi-crystalline structure. It means that the crystalline islands are surrounded by amorphous regions [39]. The radiation attack takes mainly place in amorphous areas characterized by a high disorder degree. This explains why a decrease in the crystallinity of the PCL blends is difficult to be observed. At the same time, through the formation of the crosslinks in the amorphous area, a rearrangement of the molecular chains takes place. Consequently, new nucleation centers can be formed, leading to an increase in the degree of crystallinity [36,40]. This fact could explain the observed increase of crystallinity for PCL neat, SIS:PCL= 3:1 and SIS:PCL = 1:3, respectively. A decrease of crystallinity degree for neat PCL after the exposure to 50 kGy is also observed, due to the advanced state of radiochemical degradation. The decrease in the degree of crystallinity in the samples with equal proportions of SIS and PCL can be attributed to the fact that the total amount of the amorphous zone is much smaller than in the other samples. The scission processes are predominant, and the chain rearrangement and creation of crystallization centers are prevented.

The recorded DSC curves are characterized by two oxidation maximums (OOT_1_ and OOT_2_, Figure 8), coming from the two components: 182 °C corresponding to SIS and > 270 °C corresponding to PCL, respectively. This is another proof of SIS-PCL immiscibility. The oxidation stability of all samples decreases with the irradiation dose as shown from OOT data (Table 2). The greatest decrease in OOT values noticed at 100 kGy in respect to the non-irradiated sample is observed in the case of neat PCL (~43.4 °C), while for the other samples it is only 23–24 °C. Though the initial OOT values are much higher than those obtained on any sample with SIS (e.g., 271 °C for PCL neat vs. 182 °C for SIS neat), the degradation rate of PCL is much higher in the presence of ionizing radiation. The SIS/PCL blends show OOT_1_ values comparable to those of neat SIS and the same trend of OOT decrease. This behavior suggests a possible mutual stabilizing effect between SIS and PCL. Moreover, in the case of the SIS:PCL = 1:1 sample, other features can be observed: the OOT_1_ values at doses of 25 (usually used as sterilisation dose) and 50 kGy are higher than those obtained for the other samples with SIS in the composition, at the same doses.

Regarding the second oxidation maximum (OOT_2_, corresponding to PCL), the contribution of SIS to PCL stabilization is much more obvious. The OOT_2_ values for blends are higher than in the case of neat PCL: 271.6 °C vs. 275.5 °C (SIS:PCL = 3:1), 281.5 °C (SIS:PCL = 1:3) and 290.9 °C (SIS:PCL = 1:1). This behavior could be explained by the fact that SIS, being more susceptible to oxidation, consumes oxygen from the environment through radio-induced free radicals, thus delaying the access of oxygen to PCL molecules. In other words, SIS possesses antioxidant properties for PCL.

### 3.3. FTIR Spectroscopy

FTIR analysis is a common technique used in the analysis of polymers that provides information on their chemical structure and evaluation of its state of degradation. In the case of binary systems, FTIR provides information about their miscibility or immiscibility, the blends of immiscible compounds presenting the same spectral signature as the individual components. Any interaction (of a physical or chemical nature) between the two components is easily highlighted in the FTIR spectrum (i.e., displacement, modification or disappearance/appearance of spectral characteristic bands) [41].

**Figure 10:**
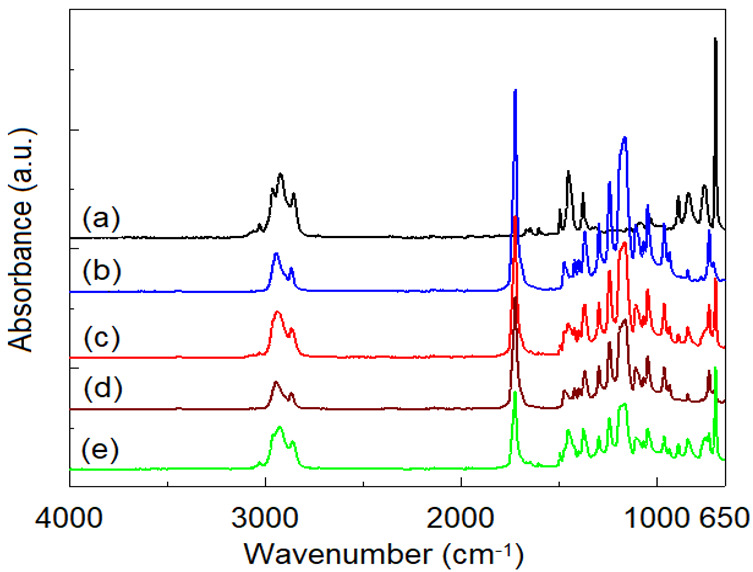
ATR/FTIR spectra recorded on: (a) SIS neat; (b) PCL neat; (c) SIS:PCL = 1:1; (d) SIS:PCL = 1:3; (e) SIS:PCL = 3:1 (Dose: 0 kGy).

**Figure 11:**
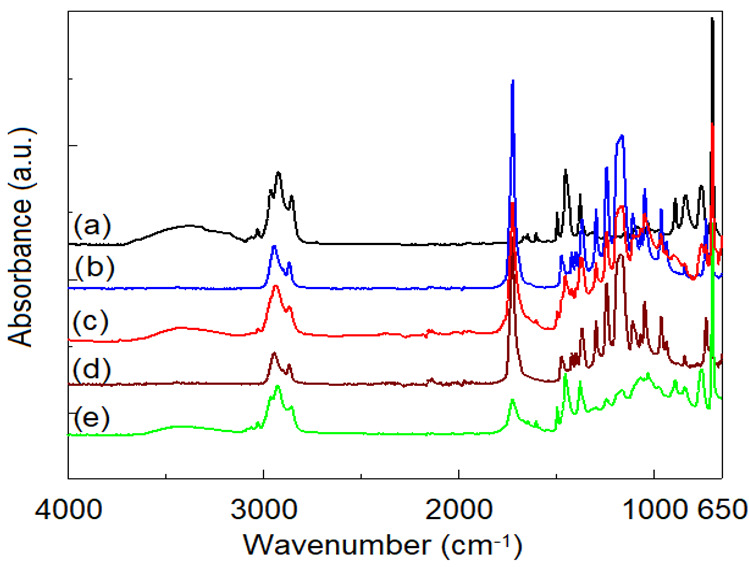
ATR/FTIR spectra recorded on: (a) SIS neat; (b) PCL neat; (c) SIS:PCL = 1:1; (d) SIS:PCL = 1:3; (e) SIS:PCL = 3:1 (Dose: 100 kGy).

Figure 10 and Figure 11 show the ATR/FTIR spectra recorded on non-irradiated and irradiated at 100 kGy samples. From the analysis of the spectra, the characteristic bands of neat SIS [9,10] can be observed at approx. 3025 cm^−1^ and 2962 cm^−1^ (corresponding to aromatic and aliphatic C-H vibrations), 1646 cm^−1^ (C = C aromatic), 1376 cm^−1^ and 1450 cm^−1^ (CH_3_ and CH_2_ bending vibrations), 888, 835 cm^−1^ (polyisoprene isomers), 758 and 698 (mono-substituted benzene), while the neat FTIR spectrum of PCL is dominated by the typical bands from 2944 and 2864 (C-H stretching), 1726 cm^−1^ (C = O stretching vibration) and 1165–1470 cm^−1^ (various deformation modes of CH_2_).

ATR/FTIR spectra recorded on SIS and PCL neat samples at different irradiation doses do not indicate significant spectral changes. However, in the case of neat SIS, a slight increase in the intensity of the band from 1736 cm^−1^ (C = O of ester type, determined from the values of the carbonyl index according to reference [28]) can be observed up to a dose of 50 kGy, after which it decreases up to 100 kGy. This fact shows the influence of the irradiation dose on the dominant process of radioinduced degradation of SIS: scission (up to 50 kGy), and crosslinking (50–100 kGy), respectively. In the case of neat PCL, in accordance with the sudden decrease in oxidation stability with irradiation dose (Table 2), the energy of gamma radiation produces a scission of any chemical bond in the PCL, chain with a preference for the —COO—CH2— bond [23,42]. Quantification of radioinduced intensity changes of certain bands is difficult to achieve from ATR/FTIR spectra. According to literature data [42], the dominant degradation process of PCL is given by radioinduced cross-linking by means of free radicals of the type —CH_2_—CH_2_—ĊH—COO— and —COO—ĊH—CH_2_—CH_2_—, with the formation of gaseous compounds such as H_2_, CO and CO_2_.

**Figure 12:**
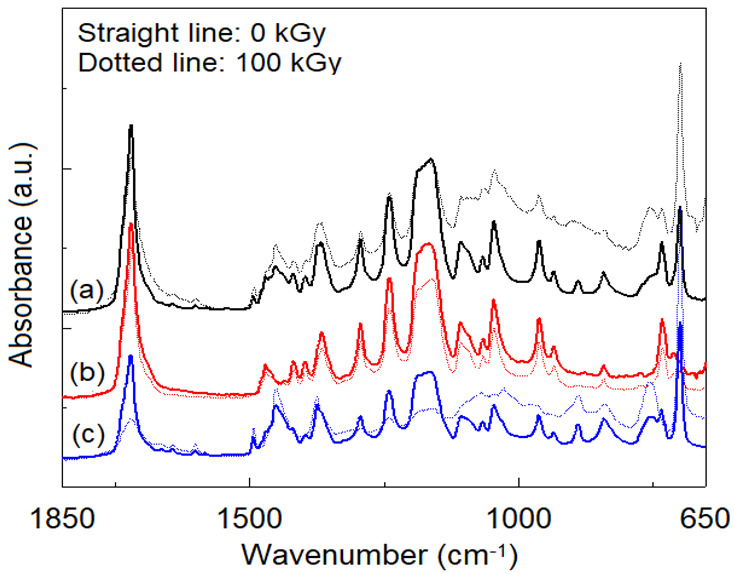
ATR/FTIR spectra recorded on non-irradiated and irradiated at 100 kGy blends: (a) SIS:PCL = 1:1; (b) SIS:PCL = 1:3; (c) SIS:PCL = 3:1.

The ATR/FTIR spectra recorded on the non-irradiated SIS:PCL blends show all the characteristic bands of the individual components, related to the concentration of the respective component, without any spectral bands displacements (Figure 12). This observation, as in the case of the DSC analysis, indicates the immiscibility of the two components. In the case of irradiation at a dose of 100 kGy, the samples with SIS in the composition present a modified spectrum in the region of the hydroxyl groups (3100–3675 cm^−1^) as an effect of radioinduced aging. Also, in the irradiated samples, a decrease in the intensity of the absorption bands in the region 1165–1470 cm^−1^ is observed, simultaneously with a decrease in the intensity of the absorption of the carbonyl band from 1726 cm^−1^. The largest spectral changes are observed in the case of SIS:PCL = 3:1 samples, followed by SIS:PCL = 1:1 and SIS:PCL = 1:3. All these spectral changes rather suggest the occurrence of interactions between the two components of the mixture than an effect of the aging of the material, suggesting the induction of a radiation-mediated compatibility between the two immiscible components.

## 4. Conclusions

This study presents the consequences caused by the radiation processing of several SIS/PCL blends. The compatibilization of components, the progress of structural and degradation modifications occurred during γ-exposures are correlated indicating the different contributions by which the fragmentation of macromolecules initiates the transformations. The chemiluminescence determinations prove that the SIS phase acts as the main source of radicals and PCL component plays the role of structural frame, while the DSC investigation reveals nucleation and the increase in the crystallinity degree. The evolution of oxidation that accompanies the radiation treatment is watched by the modification in carbonyl vibration, which demonstrates that the higher loading of SIS is favorable for the slow propagation of oxidation. Complementary, the greatest value of activation energy calculated from the isothermal CL measurements is obtained for SIS/PCL 1:1, which suggests the conclusion that this formulation is the best composition for the manufacture of engineering products by radiation processing. The greatest difference that exists between the two blended components is the nature of radicals appeared during radiolysis. While the radicals provided by SIS are centered on carbon atoms as it is occurred by hydrocarbon polymers, the radicals generated by the scission of PCL macromolecules are centered on oxygen atoms as it is happening with esters. This dissimilarity is revealed by all experimental results, which indicate the different trends of recombination for the two types of intermediates. The convenient stability of the compositions with higher content of SIS recommends them for the manufacture of various products: sealants and gaskets, protection sheets and packaging materials, toys and commodities, automotive items and many others.

## Figures and Tables

**Table 1:** The activation energies for thermal degradation of SIS/PCL systems from their OIT values.

Sample Composition	OIT * (min)			Correlation Factor	Activation Energy (kJ mol^−1^)	
	130 °C	140 °C	150 °C			
SIS	320	110	47	0.99868	136	
SIS/PCL 1:3	114	71	35	0.99184	84	
SIS/PCL 1:1	98	39	12	0.99642	148	
SIS/PCL 3:1	188	82	33	0.99917	123	

* OIT: Oxidation Induction Time.

**Table 2:** DSC parameters obtained from DSC curves analysis.

Sample	Dose (kGy)	T_m_ (°C)	ΔH_m_ (J/g)	OOT_1_ (°C)	OOT_2_ (°C)
PCL neat	0	63.2	39.0	271.6	-
	25	63.7	45.1	247.3	-
	50	63.6	49.8	241.1	-
	100	63.1	47.2	228.2	-
SIS neat	0	-	-	182.7	-
	25	-	-	159.9	-
	50	-	-	158.2	-
	100	-	-	159.6	-
SIS:PCL = 3:1	0	62.5	8.6	177.1	275.5
	25	61.9	10.4	160.3	263.2
	50	62.9	12.3	158.4	262.4
	100	61.9	14.3	154.4	254.4
SIS:PCL = 1:3	0	63.2	37.7	178.3	281.5
	25	63.3	39.2	162.3	276.6
	50	63.3	40.6	157.8	266.7
	100	63.3	42.1	154.1	264.9
SIS:PCL = 1:1	0	63.2	34.2	182.0	290.9
	25	62.9	24.1	166.5	278.8
	50	63.3	21.8	162.7	273.3
	100	63.2	17.0	158.2	265.2

## Data Availability

The data presented in this study are available on request from the corresponding authors.
